# Supplementary material for: Who gets lost and why: A representative cross-sectional survey on sociodemographic and vestibular determinants of wayfinding strategies
Source: PLoS One. 2019 Jan 30;14(1):e0204781. doi: 10.1371/journal.pone.0204781 (PMC6353538; doi:10.1371/journal.pone.0204781)
Supplement: S1 Table — (PDF) [file pone.0204781.s001.pdf]

Supporting Information

S1 Appendix. The wayfinding strategy scale as used in the questionnaire.

Wayfinding Scale Lawton 1994		Final version
Item	factor loading	Item
**Orientation Strategy**		
I keep track of the direction (north, south, east or west) in which I was going.	0.71	Ich behalte den Überblick über die Himmelsrichtung, in die ich gefahren/gegangen bin.
Before starting, I ask for directions telling me whether to go east, west, north or south at	0.7	In einer mir unbekannten Umgebung, hilft es mir zu wissen in welche Himmelsrichtung ich fahren/gehen soll.
I keep track of where I am in relationship to the sun (or moon) in the sky as I go.	0.64	Ich behalte ich den Überblick darüber, wo ich in Bezug zur Sonne beziehungsweise zum Mond bin.
I keep track of the relationship between where I am and the center of town.	0.62	Ich behalte den Überblick darüber, wo ich mich im Bezug zur Stadtmitte befinde
As I drive, I make a mental note of the mileage I travel on different roads.	0.56	Während ich fahre/gehe, merke ich mir, wie weit ich auf den verschiedenen Straßen schon gefahren/gegangen bin.
Before starting, I ask for directions telling me how far to go in terms of mileage.	0.55	In einer mir unbekannten Umgebung, hilft es mir zu wissen, wie weit ich von meinem Ziel entfernt bin.
I keep track of the relationship between where I am and the next place where I have to change direction.	0.46	Ich behalte den Überblick darüber, wann ich links und rechts abbiegen muss.
I visualize a map or layout of the area in my mind as I drive.	0.41	Ich stelle mir eine Karte oder den Grundriss der Gegend beim Fahren/Gehen bildlich vor.
I refer to a published road map when I drive (gelöscht)	0.4	deleted
**Route Strategy**		
Before starting, I ask for directions telling me whether to turn right or left at particular streets or landmarks.	0.8	In einer mir unbekannten Umgebung, hilft es mir zu wissen, an welchen Straßen oder Orientierungspunkten ich links oder rechts abbiegen muss.

Wayfinding Scale Lawton 1994		Final version
Before starting, I ask for directions telling me how many streets to pass before making each turn.	0.77	In einer mir unbekannten Umgebung, hilft es mir zu wissen, wie viele Seitenstraßen ich passieren muss, bevor ich jeweils abbiege.
As I drive, I make a mental note of the number of streets I pass before making each turn.	0.59	Wenn ich fahre/gehe, merke ich mir die Anzahl der Seitenstraßen, bevor ich jeweils abbiege.
Before starting, I ask for a hand-drawn map of the area	0.57	deleted
I make a mental note of landmarks, such as buildings or natural features, that I pass along the	0.42	Ich merke mir auf meinem Weg Orientierungspunkte, wie z.B. Gebäude oder natürliche Landschaftsmerkmale.
I found maps of the building or complex, with an arrow pointing to may present location, to be very helpful (new)		In einer mir unbekannten Umgebung, hilft es mir zu wissen, an welchem Punkt auf der Karte ich mich momentan befinde (neu)

Example:

	Individual total sum scores from example	Max attainable scores	Individual percentage sum scores	scores scaled to 100
orientation strategy	3+2+2+4+4+3+4+4=26	5*8=40	26/40=0.65	65
route strategy	2+2+3+3+2=12	5*5=25	12/25=0.48	48
