# Supplementary material for: Who gets lost and why: A representative cross-sectional survey on sociodemographic and vestibular determinants of wayfinding strategies
Source: PLoS One. 2019 Jan 30;14(1):e0204781. doi: 10.1371/journal.pone.0204781 (PMC6353538; doi:10.1371/journal.pone.0204781)
Supplement: S2 Table — (metric). (PDF) [file pone.0204781.s002.pdf]

S2 Appendix. Alternative linear regression models, including lin-log regression and age in yrs. (metric)

Alternative linear regression models for orientation strategy, weighted according to frequency distribution of the current population by individual weighting factors, n=783

		Model 1: all			Model 2: without income			Model 3: interaction			Model 4: age metric			Model 5:lin -log			Model 6:lin-log interaction		
	model fit	R^2=0.098 F=6.316***			R^2=0.098 F=7.502***			R^2=0.097 F=5.414***			R^2=0.094 F=6.398***			R^2=0.098 F=6.329***			R^2=0.098 F=5.478***		
		ß1	95%-CI	p	ß1	95%-CI	P	ß1	95%-CI	p	ß1	95%-CI	p	ß1	95%-CI	p	ß1	95%-CI	p
	Intercept	53.47	48.74; 58.2	0.00	52.56	48.26; 56.86	0.00	53.70	48.76; 58.65	0.00	51.28	44.67; 57.89	0.00	3.92	3.83; 4	0.00	3.91	3.82; 4	0.00
	gender (ref:women)	4.68	2.05; 7.31	0.00	4.54	1.92; 7.16	0.00	4.71	-0.39; 9.8	0.07	4.85	-2.78; 12.48	0.21	0.10	0.05; 0.15	0.00	0.13	0.04; 0.22	0.01
age in years. classes (ref:18-35)	36-55	4.21	0.9; 7.53	0.01	3.96	0.68; 7.24	0.02	3.94	-0.38; 8.25	0.07	-	-	-	0.08	0.02; 0.14	0.00	0.09	0.01; 0.17	0.02
	56-70	2.40	-1.51; 6.31	0.23	2.10	-1.77; 5.98	0.29	4.22	-1.23; 9.67	0.13	-	-	-	0.04	-0.03; 0.11	0.30	0.09	-0.01; 0.19	0.06
	71-96	7.31	2.92; 11.7	0.00	7.35	2.98; 11.73	0.00	5.99	0.38; 11.6	0.04	-	-	-	0.12	0.04; 0.2	0.00	0.11	0.01; 0.22	0.03
age-gender-interaction	men*(36-55)	-	-	-	-		-	0.59	-5.97; 7.15	0.86	-	-	-	-	-	-	-0.03	-0.15; 0.09	0.66
	men*(56-70)	-	-	-	-		-	-3.12	-10.8; 4.56	0.43	-	-	-	-	-	-	-0.11	-0.25; 0.03	0.13
	men*(71-96)	-	-	-	-		-	2.93	-5.52; 11.38	0.50	-	-	-	-	-	-	0.00	-0.15; 0.16	0.97
	age (metric)	-	-	-	-		-	-	-	-	0.11	0.01; 0.21	0.03	-	-	-	-		-
	age-gender-interaction	-	-	-	-		-	-	-	-	0.00	-0.15; 0.14	0.97	-	-	-	-		-
education (ref: primary education)	secondary. non-tertiary	3.81	0.61; 7.02	0.02	3.66	0.5; 6.82	0.02	3.65	0.4; 6.9	0.03	3.96	0.7; 7.23	0.02	0.08	0.02; 0.13	0.01	0.07	0.01; 0.13	0.02
	upper secondary	5.02	0.51; 9.52	0.03	4.63	0.18; 9.09	0.04	4.91	0.34; 9.47	0.04	5.02	0.46; 9.58	0.03	0.10	0.02; 0.18	0.02	0.09	0.01; 0.18	0.03
	tertiary	11.48	7.39; 15.58	0.00	10.85	6.93; 14.76	0.00	11.32	7.22; 15.43	0.00	11.45	7.33; 15.57	0.00	0.21	0.14; 0.29	0.00	0.21	0.14; 0.28	0.00
net incomein €,classes (ref:>1.500	1.500 up to 2.500	-1.89	-5.41; 1.64	0.29	-	-	-	0.22	-5.82; 1.33	0.40	0.21	-5.77; 1.25	- 0.13	0.34	-0.1; 0.03	- 0.13	0.26	-0.1; 0.03	0.26
	2.5000 up to 3.500	-0.09	-4; 3.83	0.97	-	-	-	-0.23	-4.17; 3.71	0.91	-0.41	-4.27; 3.46	0.84	- 0.02	-0.09; 0.05	0.55	-0.02	-0.09; 0.05	0.56
	>3.500€	-3.06	-7.12; 1	0.14	-	-	-	-3.12	-7.19; 0.96	0.13	-3.19	-7.17; 0.8	0.12	- 0.07	-0.14; 0.01	0.08	-0.07	-0.14; 0.01	0.07
regions (ref: >50.000)	50.000 up to 100.000	-0.14	-4.79; 4.51	0.95	-0.07	-4.71; 4.58	0.98	-0.23	-4.89; 4.43	0.92	-0.03	-4.68; 4.63	0.91	0.01	-0.08; 0.09	0.82	0.01	-0.08; 0.09	0.87
	100.000 up to 500.000	1.07	-2.47; 4.61	0.55	0.74	-2.79; 4.27	0.68	1.10	-2.46; 4.65	0.55	0.88	-2.67; 4.42	0.63	0.02	-0.05; 0.08	0.57	0.02	-0.05; 0.08	0.55
	500.000 and more	-4.38	-7.85; -0.91	0.01	-4.35	-7.82; -0.87	0.01	-4.40	-7.87; -0.92	0.01	-4.38	-7.86; -0.91	0.01	- 0.08	-0.14; -0.02	0.02	-0.08	-0.14; -0.02	0.02
	lifetime prevalence of vertigo (ref:no)	-6.10	-9.34; -2.86	0.00	-6.43	-8.96; -2.53	0.00	-5.95	-9.21; -2.7	0.00	-6.05	-9.29; -2.81	0.00	- 0.10	-0.16; -0.04	0.00	-0.10	-0.16; -0.04	0.00
balance (ref:equal)	worse	2.92	-2.03; 7.86	0.25	2.73	-2.21; 7.67	0.28	3.04	-1.92; 7.99	0.23	2.74	-2.24; 7.72	0.28	0.05	-0.04; 0.14	0.31	0.05	-0.04; 0.14	0.28
	better	4.54	1.76; 7.31	0.00	4.55	1.79; 7.31	0.00	4.46	1.65; 7.27	0.00	4.48	1.69; 7.28	0.00	0.08	0.03; 0.13	0.00	0.08	0.02; 0.13	0.00

Alternative linear regression models for route strategy, weighted according to frequency distribution of the current population by individual weighting factors, n=783

		Model 1: all			Model 2: without income			Model 3: interaction			Model 4: age metric			Model 5:lin -log			Model 6:lin-log interaction		
model fit		R^2=0.081 F=5.299***			R^2=0.081 F=6.286***			R^2=0.096 F=5.394***			R^2=0.069 F=4.891***			R^2=0.082 F=5.364***			R^2=0.098 F=5.489***		
		ß1	95%-CI	p	ß1	95%-CI	p	ß1	95%-CI	p	ß1	95%-CI	p	ß1	95%-CI	p	ß1	95%-CI	p
	Intercept	65.83	60.89; 70.76	0.00	67.11	62.62; 71.6	0.00	66.85	61.74; 71.96	0.00	68.53	61.61; 75.45	0.00	4.15	4.07; 4.24	0.00	4.17	4.08; 4.25	0.00
	gender (ref:women)	2.95	0.2; 5.69	0.04	3.04	0.3; 5.77	0.03	-0.12	-5.38; 5.15	0.97	5.71	-2.28; 13.7	0.16	0.05	0; 0.09	0.07	0.00	-0.09; 0.09	0.99
age in years. classes (ref:18-35)	36-55	3.34	-7.53; 1.6	0.06	3.51	0.09; 6.93	0.04	-0.83	-5.29; 3.64	0.72				0.02	-0.04; 0.08	0.46	-0.05	-0.13; 0.03	0.22
	56-70	-2.69	-6.77; 1.39	0.12	-2.40	-6.44; 1.64	0.24	0.65	-4.99; 6.28	0.82				-0.07	-0.14; 0	0.06	-0.01	-0.1; 0.09	0.93
	71-96	-2.81	-7.39; 1.77	0.23	-2.97	-7.53; 1.6	0.20	-4.05	-9.85; 1.75	0.17				-0.07	-0.15; 0.01	0.08	-0.08	-0.18; 0.02	0.12
age-gender-interaction	geschl*alter(36-55)							9.41	2.63; 16.19	0.01							0.16	0.04; 0.28	0.01
	geschl*alter(56-70)							-4.57	-12.5; 3.37	0.26							-0.09	-0.23; 0.05	0.19
	geschl*alter(71-96)							3.18	-5.56; 11.91	0.48							0.03	-0.12; 0.18	0.72
	age (metric)										-0.05	-0.15; 0.06	0.40						
	age-gender-interaction										-0.06	-0.21; 0.09	0.44						
education (ref: primary education)	secondary. non-tertiary	4.78	1.44; 8.12	0.01	4.92	1.63; 8.21	0.00	4.54	1.18; 7.9	0.01	4.60	1.18; 8.02	0.01	0.09	0.03; 0.15	0.00	0.08	0.03; 0.14	0.01
	upper secondary	8.92	4.22; 13.62	0.00	8.80	4.15; 13.45	0.00	9.23	4.51; 13.94	0.00	7.83	3.06; 12.6	0.00	0.15	0.07; 0.23	0.00	0.16	0.08; 0.24	0.00
	tertiary	9.70	5.43; 13.97	0.00	9.72	5.64; 13.8	0.00	9.46	5.22; 13.7	0.00	9.45	5.14; 13.77	0.00	0.17	0.1; 0.24	0.00	0.17	0.09; 0.24	0.00
net income in €. classes (ref:>500t)	1.500 up to 2.500	1.79	-1.89; 5.46	0.34				1.40	-2.3; 5.09	0.46	2.00	-1.67; 5.68	0.29	0.05	-0.02; 0.11	0.17	0.04	-0.02; 0.1	0.23
	2.5000 up to 3.500	3.23	-0.85; 7.31	0.12				2.75	-1.32; 6.82	0.19	4.13	0.08; 8.17	0.05	0.07	-0.01; 0.14	0.07	0.06	-0.01; 0.13	0.12
	>3.500€	0.56	-3.68; 4.79	0.80				0.85	-3.36; 5.06	0.69	1.45	-2.72; 5.62	0.50	0.02	-0.06; 0.09	0.66	0.02	-0.05; 0.09	0.56
regions (ref: >50.000)	50.000 up to 100.000	-0.55	-5.4; 4.3	0.82	-0.35	-5.19; 4.49	0.89	-0.30	-5.12; 4.51	0.90	-0.44	-5.31; 4.44	0.86	0.00	-0.09 0.08	0.94	0.00	-0.08; 0.09	0.97
	100.000 up to 500.000	-1.58	-5.28; 2.11	0.40	-1.63	-5.3; 2.05	0.39	-1.05	-4.73; 2.63	0.58	-1.86	-5.57; 1.85	0.33	-0.03	-0.1; 0.03	0.30	-0.02	-0.09; 0.04	0.46
	500.000 and more	-1.31	-4.94; 2.31	0.48	-1.32	-4.94; 2.3	0.47	-1.31	-4.91; 2.28	0.47	-1.57	-5.21; 2.07	0.40	-0.04	-0.11; 0.02	0.17	-0.04	-0.11; 0.02	0.17
	lifetime prevalence of vertigo (ref:no)	-4.73	-8.12; -1.35	0.01	-4.73	-8.09; -1.38	0.01	-4.22	-7.59; -0.86	0.01	-5.01	-8.4; -1.61	0.00	-0.07	-0.13; -0.02	0.01	-0.07	-0.12; -0.01	0.03
balance (ref:equal)	worse	0.19	-4.96; 5.35	0.94	0.18	-4.97; 5.33	0.95	0.64	-4.47; 5.76	0.81	0.43	-4.78; 5.64	0.87	-0.05	-0.14; 0.04	0.31	-0.04	-0.13; 0.05	0.40
	better	2.52	-0.37; 5.42	0.09	2.40	-0.47; 5.28	0.10	2.57	-0.34; 5.47	0.08	2.22	-0.7; 5.15	0.14	0.03	-0.02; 0.08	0.21	0.03	-0.02; 0.08	0.21

Model equations

Model 1	y=b0+b1*Dgender+b2*Dage+b3*Deducation+b4*Dincome+b5*Dregion+b6*Dvertigo+b7*Dbalance
Model 2	y=b0+b1*Dgender+b2*Dage+b3*Deducation+b4*Dregion+b6*Dvertigo+b7*Dbalance
Model 3	y=b0+b1*Dgender+b2*Dage+b3*Deducation+b4*Dincome+b5*Dregion+b6*Dvertigo+b7*Dbalance+b8*Dage_gender
Model 4	y=b0+b1*Dgender+b2*age+b3*Deducation+b4*Dincome+b5*Dregion+b6*Dvertigo+b7*Dbalance+b8*age_gender
Model 5	ln(y)=b0+b1*Dgender+b2*Dage+b3*Deducation+b4*Dincome+b5*Dregion+b6*Dvertigo+b7*Dbalance
Model 6	ln(y)=b0+b1*Dgender+b2*Dage+b3*Deducation+b4*Dincome+b5*Dregion+b6*Dvertigo+b7*Dbalance+b8*Dage_gender
