# Supplementary material for: Who gets lost and why: A representative cross-sectional survey on sociodemographic and vestibular determinants of wayfinding strategies
Source: PLoS One. 2019 Jan 30;14(1):e0204781. doi: 10.1371/journal.pone.0204781 (PMC6353538; doi:10.1371/journal.pone.0204781)
Supplement: S3 Table — (PDF) [file pone.0204781.s003.pdf]

Appendix 2. Combination in the multinomial regression model (n = 783). Odds ratios (OR), Confidence Intervals (CI) and p-values (p). Outcomes were individually weighted according to frequency distribution of the current population. OR >1 means it is more likely to be part of the group of interest.

		Odds(Both strategies:none strategy)				Odss (orientation strategy: route strategy)				Odds (orientation strategy: both strategies)			
		Exp(B)	CI:lower limit	CI: upper limit	p	Exp(B)	CI:lower limit	CI: upper limit	p	Exp(B)	CI:lower limit	CI: upper limit	p
gender (ref: woman)		1.75	1.19	2.58	0.01	1.72	1.00	2.95	0.05	1.32	0.82	2.13	0.26
Age (ref: 18-35)	36-55	2.35	1.42	3.89	0.00	0.26	0.13	0.52	0.00	0.22	0.11	0.41	0.00
	56-70	1.31	0.74	2.32	0.36	0.64	0.29	1.42	0.27	0.42	0.21	0.84	0.01
	71-96	1.78	0.92	3.46	0.09	2.54	1.00	6.45	0.05	0.87	0.42	1.78	0.70
education[1] (ref: primary)	secondary	1.49	0.94	2.36	0.09	0.92	0.48	1.76	0.80	0.72	0.41	1.27	0.26
	upper 2nd	3.04	1.55	5.98	0.00	0.25	0.09	0.65	0.01	0.22	0.09	0.55	0.00
	tertiary	4.58	2.44	8.60	0.00	0.62	0.26	1.48	0.28	0.27	0.12	0.58	0.00
income (ref: lowest)	2nd lowest	0.54	0.32	0.91	0.02	0.94	0.46	1.90	0.85	1.48	0.80	2.74	0.22
	2nd highest	0.85	0.48	1.49	0.56	0.86	0.38	1.97	0.73	0.83	0.41	1.70	0.62
	highest	0.63	0.35	1.16	0.14	1.72	1.00	2.95	0.05	1.21	0.59	2.52	0.60
bik-region[2] (>20.000)	20-50k	0.76	0.38	1.53	0.45	1.00	0.38	2.58	0.99	2.31	0.94	5.67	0.07
	50-100k	0.94	0.57	1.57	0.82	3.32	1.51	7.29	0.00	2.90	1.48	5.70	0.00
	<500k	1.15	0.70	1.88	0.59	1.08	0.50	2.35	0.84	1.68	0.84	3.39	0.14
sense of balance (ref: same)	worse	1.74	0.81	3.75	0.16	0.82	0.46	1.47	0.52	1.33	0.55	3.22	0.52
	better	1.90	1.26	2.86	0.00	0.94	0.37	2.36	0.90	0.61	0.37	1.01	0.06
vertigo (ref: no)	yes	2.57	1.59	4.14	0.00	1.78	0.89	3.57	0.10	1.04	0.55	1.99	0.90

		Odds (route strategy: both strategies)				Odds (orientation strategy: none strategy)				Odds (route strategy: none strategy)			
		Exp(B)	CI:lower limit	CI: upper limit	p	Exp(B)	CI:lower limit	CI: upper limit	p	Exp(B)	CI:lower limit	CI: upper limit	p
gender (ref: woman)		0.77	0.50	1.18	0.22	2.30	1.41	3.75	0.00	1.34	0.85	2.11	0.21
Age (ref: 18-35)	36-55	0.83	0.49	1.43	0.51	0.51	0.27	0.98	0.04	1.96	1.12	3.44	0.02
	56-70	0.65	0.33	1.30	0.22	0.55	0.28	1.08	0.08	0.85	0.43	1.68	0.65
	71-96	0.34	0.14	0.82	0.02	1.54	0.75	3.15	0.24	0.61	0.25	1.46	0.27
education[1] (ref: primary)	secondary	0.78	0.45	1.37	0.39	1.07	0.61	1.87	0.81	1.17	0.68	2.01	0.58
	upper 2nd	0.89	0.44	1.81	0.75	0.66	0.26	1.69	0.39	2.71	1.29	5.71	0.01
	tertiary	0.43	0.22	0.85	0.02	1.22	0.54	2.80	0.63	1.99	0.95	4.17	0.07
income (ref: lowest)	2nd lowest	1.58	0.86	2.92	0.14	0.79	0.43	1.46	0.46	0.85	0.46	1.56	0.60
	2nd highest	0.97	0.50	1.87	0.92	0.71	0.34	1.48	0.36	0.82	0.41	1.63	0.57
	highest	0.77	0.50	1.18	0.22	2.30	1.41	3.75	0.00	1.16	0.58	2.33	0.68
bik-region[2] (>20.000)	20k-50k	2.32	1.10	4.90	0.03	1.76	0.73	4.26	0.21	1.77	0.84	3.74	0.14
	50k-100k	0.87	0.47	1.64	0.68	2.74	1.40	5.34	0.00	0.82	0.44	1.56	0.55
	<500k	1.56	0.88	2.75	0.13	1.93	0.95	3.90	0.07	1.78	0.99	3.21	0.05
sense of balance (ref: same)	worse	0.74	0.47	1.18	0.20	1.16	0.69	1.96	0.58	1.41	0.86	2.30	0.17
	better	1.42	0.63	3.19	0.40	2.32	0.99	5.46	0.05	2.47	1.12	5.45	0.03
vertigo (ref: no)	yes	0.59	0.34	1.02	0.06	2.68	1.45	4.96	0.00	1.50	0.88	2.56	0.13

[1] Categorization of German academic achievement according to ISCED 1997:
Primary education/lower secondary education=Volks-/Hauptschulabschluss
Lower Secondary education=German Realschulabschluss and further education without diploma "Abitur" as well as current students/pupils
Upper secondary education=Abitur/(Fach)hochschulreife
Tertiary education=diploma for university and higher degree
[2] BIK-regions=measure of regional urbanization and are classified by the number of inhabitants
